# Effects of Soft Denture Liners on L929 Fibroblasts, HaCaT Keratinocytes, and RAW 264.7 Macrophages

**DOI:** 10.1155/2014/840613

**Published:** 2014-09-11

**Authors:** Carolina de Andrade Lima Chaves, Carlos Alberto de Souza Costa, Carlos Eduardo Vergani, Pedro Paulo Chaves de Souza, Ana Lucia Machado

**Affiliations:** ^1^Department of Dental Materials and Prosthodontics, Araraquara Dental School, Universidade Estadual Paulista (UNESP), Rua Humaitá 1680, 14801-903 Araraquara, SP, Brazil; ^2^Department of Physiology and Pathology, Araraquara Dental School, Universidade Estadual Paulista (UNESP), Rua Humaitá 1680, 14801-903 Araraquara, SP, Brazil

## Abstract

The effects of six soft liners (Ufi Gel P (UG), Sofreliner S (SR), Durabase Soft (D), Trusoft (T), Coe Comfort (CC), and Softone (ST)) on L929, HaCat, and RAW 264.7 cells were investigated. Eluates (24 and 48 h) from the materials were applied on the cells and the viability, type of cell death, and morphology were evaluated. Cells were also seeded on the specimens' surfaces (direct contact) and incubated (24 or 48 h), and viability was analyzed. Controls were cells in culture medium without eluates or specimens. For cell viability, no significant differences were found among materials or between extraction periods, and the liners were noncytotoxic or slightly cytotoxic. Morphology of RAW 264.7 cells was altered by the 24 h eluates from CC and D and the 48 h eluates from SR, CC, and D. The 24 and 48 h eluates from all materials (except T) increased the percentages of L929 necrotic cells. For direct contact tests, the lowest cytotoxicity was observed for UG and SR. Although eluates did not reduce viability, morphology alterations and increase in necrosis were seen. Moreover, in the direct contact, effects on viability were more pronounced, particularly for D, T, CC and ST. Thus, the use of UG and SR might reduce the risk of adverse effects.

## 1. Introduction

Soft reline materials are often used to provide better fit and comfort for patients who cannot tolerate conventional hard-denture bases because of excessive residual ridge resorption, bruxism, xerostomia, and fragile supporting mucosa [1]. The soft liners based on acrylic resins are composed of a powder (polyethylmethacrylate) and a liquid containing monomer and plasticizer (phthalate esters) [1, 2]. For the silicone-based liners [3], the polymer is an elastomer (polydimethylsiloxane) that does not require an external plasticizer and, therefore, is more stable over time [2]. Other soft liners have been developed specifically as tissue conditioners [1, 4]. In these materials, the mixture of a polyethylmethacrylate powder and the liquid containing phthalates, ethanol, and no monomer results in the formation of a soft gel which readily flows to adapt to the supporting tissues [1, 5, 6].

Both soft liners and tissue conditioners may release components as residual monomers, plasticizers, degradation products [7, 8], and alcohol [3, 4]. Substances released from these materials into saliva can then diffuse across the oral or gastrointestinal mucosa, causing adverse reactions [9–11]. Thus, the biocompatibility of soft liners and tissue conditioners has been evaluated through tests involving exposure of the cells to substances released (eluates) from such materials [5, 12–14]. However, scarce data concerning the cytotoxicity of soft liners are available.

Only a few studies evaluated the effects of direct contact between cells and soft liners [15–18]. This is particularly important since these materials have also been used extensively in areas of ulceration and inflamed tissues caused by poorly fitting dentures [1], in patients who have diabetes or other debilitating diseases [19], for aftercare of immediate dentures, or during the osseointegration of dental implants [20]. Therefore, the aim of this study was to evaluate* in vitro* the effects of four soft reline materials (two silicone-based and two acrylic-based) and two tissue conditioners on different cell lines (L929, HaCat, and RAW 264.7), by analysis of cell viability (MTT assay), cell morphology (SEM), and patterns of cell death (flow cytometry). The assays were performed after the cells were either exposed to the eluates from the materials or in direct contact with the materials for 24 or 48 h.

## 2. Materials and Methods

### 2.1. Materials

The materials selected for this study, codes, types, lot numbers, manufacturers, compositions, and powder/liquid proportions, are presented in Table 1.

The cytotoxicity tests were conducted according to ISO 10993-5 [21]. The specimens (10 × 1 mm) of each material were prepared under aseptic conditions [13]. The materials were mixed according to the manufacturers' instructions and inserted into metal molds; pressure was applied until the reaction was complete.

### 2.2. Eluate Preparation

Extracts were obtained by incubating the specimens of each material in culture medium for either 24 or 48 h [12]. They were individually placed on 24-well plates with 3 mL of Dulbecco's modified Eagle medium (DMEM—Sigma Chemical Co., St. Louis, MO, USA) with antibiotics (Gibco, Grand Island, NY, USA) in each well and incubated at 37°C with 5% CO_2_ and 95% air for 24 or 48 h. Culture medium without test specimens was also incubated under the same conditions and served as control.

### 2.3. Cell Culture

The three immortalized cell lines, L929 fibroblasts, human keratinocytes (HaCaT-CLS 300493), and macrophage RAW 264.7, used in this study were cultivated in the Laboratory of Experimental Pathology and Biomaterials, Araraquara Dental School, Brazil. The cell lines were grown in Dulbecco's modified Eagle medium (Sigma Chemical Co.) supplemented with 10% fetal bovine serum, 100 IU/mL penicillin, 100 *μ*g/mL streptamycin, and 2 mmol/L glutamine (Gibco). The cultures were maintained in 75 cm^2^ culture flasks at 37°C in a humidified 5% CO_2_ incubator with routine passage every 3 days until adequate numbers of cells were obtained, when they were seeded in sterile culture plates.

### 2.4. Analysis of Cellular Metabolism

After reaching approximately 80% cell density, the cells were trypsinized, seeded in sterile 96-well plates (1 × 10^4^ cells/mL), and incubated for 24 h, after which medium containing the 24 or 48 hour eluates (100 *μ*L) from the materials was added to each well of the 96-well culture plate and incubated for another 24 h at 37°C with 5% CO_2_ and 95% air. In the same experimental conditions, wells containing cells and fresh culture medium were used as control.

The effects of the soft liners and tissue conditioners on cellular metabolism were also assessed after direct contact of the cells with the materials. For this, the specimens of each material were placed in 24-well plates, and the cells were seeded (1 × 10^5^ cells/mL) on the surfaces of the specimens [21] and incubated at 37°C with 5% CO_2_ and 95% air for 24 or 48 h.

For the MTT assay, the culture medium was aspirated, and a 100 *μ*L quantity of MTT solution (5 mg/mL in phosphate-buffered saline) (Sigma Chemical Co.) and a 900 *μ*L quantity of DMEM without bovine serum were added to each well and incubated at 37°C for 4 h. Thereafter, the culture medium with MTT solution was aspirated, and the formazan crystals, resulting from the cleavage of the MTT salt ring by the succinic dehydrogenase (SDH) enzyme present in the mitochondria of viable cells, were solubilized with acidified isopropanol solution (0.04 N HCl). An ELISA plate reader (BIO-RAD, model 3550-UV, Hercules, CA, USA) was used to assess cell viability as being proportional to the absorbance determined by spectrophotometry at a 570 nm wavelength. For each experimental condition, the mean values of two aliquots were averaged to provide a single value. Results of MTT assay were expressed as percentage of controls (cells in medium without the eluates or test specimens), which was considered as 100%.

The tests with eluates or direct contact were performed on at least three separate occasions. In each occasion, 6 specimens of each material and control were prepared (4 were used in the MTT assay and 2 in the analysis of cell morphology, as described below).

### 2.5. Analysis of Cell Morphology by Scanning Electron Microscopy (SEM)

Sterile glass coverslips (12 mm) (Fisher Scientific, Atlanta, GA) were placed on the bottoms of the experimental (cells exposed to the 24 or 48 hour eluates) and control wells, immediately before the cells were seeded. After 24 h of incubation, the culture medium was removed, and the viable cells that still adhered to the glass coverslips were fixed in 1 mL of 2.5% glutaraldehyde for 2 h. Subsequently, the coverslips were washed with PBS and distilled water, and the cells were dehydrated with ethanol solutions in ascending concentrations (30%, 50%, 70%, 95%, and 100%) and immersed in 1,1,1,3,3,3-hexamethyldisilazane (Acros Organics, Springfield, NJ, USA) for 90 min. The coverslips were fixed on metal stubs and placed in a desiccator for 7 days then sputter-coated with gold, and the morphology of the surface-adhered cells was examined by scanning electron microscopy (FEI Inspect S50, Company, Brno, Czech Republic).

### 2.6. Analysis by Flow Cytometry with Annexin-V and Propidium Iodide

Annexin-V is a protein that possesses high affinity for phosphatidylserine, a lipid that is translocated to the outer surface of the membrane and exposed to the extracellular milieu, in cells undergoing apoptosis. Propidium iodide is a fluorescent dye that is able to bind to DNA only when the membrane is damaged, thus identifying the cells killed by necrosis. Flow cytometry analysis was performed with the cell lines L929 and HaCat, after exposure to the 24 or 48 hour eluates from the materials. Types of cell death were analyzed with a FACSCalibur flow cytometer equipped with an argon laser and CellQuest software (Becton Dickinson, Franklin Lakes, NJ, USA). For each experimental condition and controls, at least 10,000 events were collected and analyzed. After incubation of cells in 24-well plates (1 × 10^5^ cells/mL) for 24 h, the culture medium was removed and the cells were incubated with the 24 or 48 h eluates (*n* = 8 per time period, i.e., *n* = 16 in total per material) obtained from the materials. Of these, 4 were analyzed for FACS on two separate occasions. Cells used for controls (culture medium without eluates) were incubated under the same conditions (*n* = 8 per time period, i.e., *n* = 16 in total). The cells were then trypsinized and centrifuged at 500 rpm for 5 min. The supernatant was discarded and the cells were resuspended in 300 *μ*L binding buffer with 10 mM HEPES (pH 7.4, comprised of 150 mM NaCl, 5 mM KCl, 1 mM MgCl_2_, and 1.8 mM CaCl_2_). For acquisition of the cells labeled positively for apoptosis, a 250 *μ*L aliquot of the cell suspension of each condition (experimental and control) was treated with Annexin-V (Institute of Biomedical Sciences, São Paulo, SP, Brazil) at a concentration of 1 : 500 for 20 min in the dark. The apoptotic cells were acquired in the FL-1 channel of the flow cytometer. The acquisition of cells labeled positively for necrosis was performed immediately after the addition of 0.2 *μ*g/mL propidium iodide to the FL-2 channel of the flow cytometer.

### 2.7. Statistical Analysis

All statistical analyses were performed with SPSS for Windows (release 17.0, SPSS Inc., Chicago, IL, USA). Data from MTT tests (with eluates and direct contact) and from flow cytometry were analyzed by two-way ANOVA followed by Tukey's test, with *P* ≤ 0.05. Data from flow cytometry were log transformed to comply with ANOVA assumptions.

## 3. Results

### 3.1. Effects of the Eluates from the Soft Liner Materials on Cell Viability, Cell Morphology, and Type of Cell Death

Means and standard deviations of the percentages of cell viability relative to controls, obtained in the MTT assay, are shown in Table 2. For L929 and RAW 264.7 cells, no significant differences were found among materials or between the two extraction periods. For HaCat cells, exposure to the 48 hour eluates resulted in significantly lower percentages of cell viability than those obtained with the 24 hour eluates, regardless of the materials.

**Figure 1:**
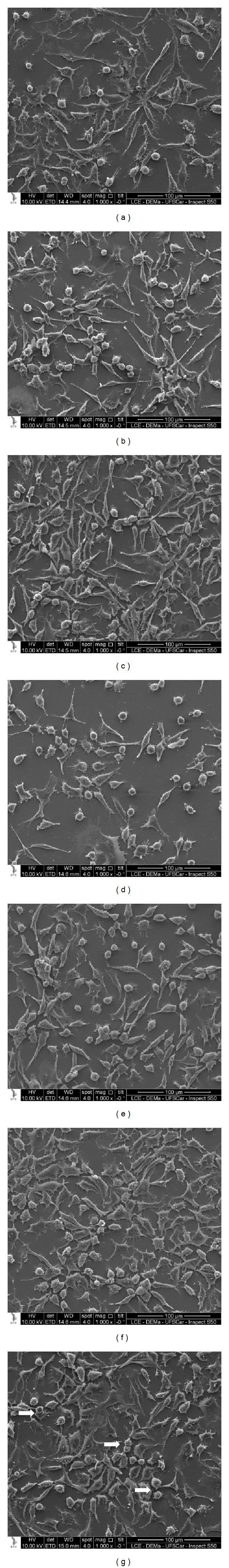
Panel of SEM micrographs (×1000) representative of the L929 cells exposed to the 24 hour eluates from materials (a) Ufi Gel P, (b) Sofreliner S, (c) Coe Comfort, (d) Softone, (e) Durabase Soft, (f) Trusoft, and (g) control (medium without eluate).

**Figure 2:**
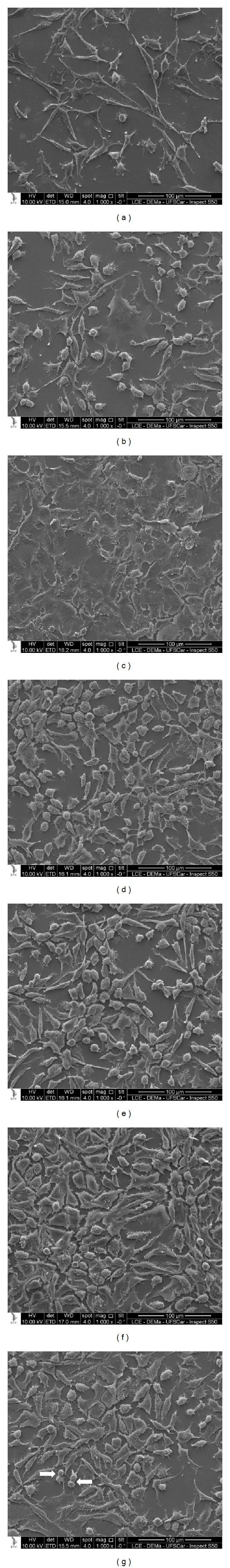
Panel of SEM micrographs (×1000) representative of the L929 cells exposed to the 48 hour eluates from materials (a) Ufi Gel P, (b) Sofreliner S, (c) Coe Comfort, (d) Softone, (e) Durabase Soft, (f) Trusoft, and (g) control (medium without eluate).

Figures 1 and 2 show the photomicrographs of L929 cell for controls (cells exposed to medium without eluate) and experimental conditions (exposure to the 24 and 48 hour eluates from the soft liner materials). As shown in Figures 1(g) and 2(g), control cells displayed their characteristic spindle-shaped morphology, covering the glass substrate and undergoing mitosis. No significant changes in morphology occurred after incubation with the 24 and 48 hour eluates from all materials (Figures 1(a)–1(f) and Figures 2(a)–2(f)).

**Figure 3:**
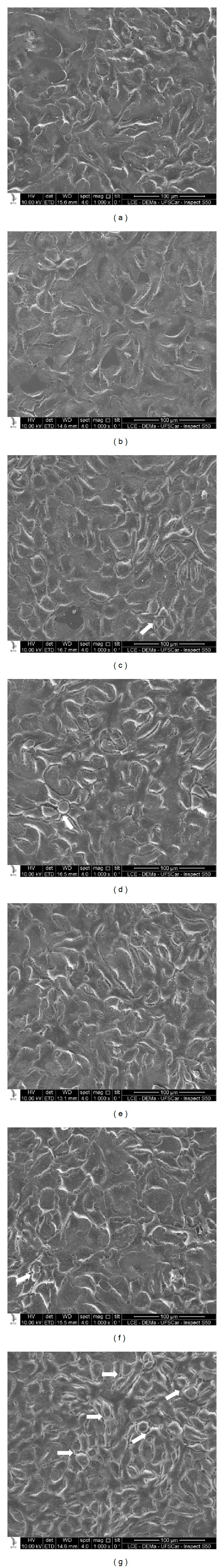
Panel of SEM micrographs (×1000) representative of the HaCat cells exposed to the 24 hour eluates from materials (a) Ufi Gel P, (b) Sofreliner S, (c) Coe Comfort, (d) Softone, (e) Durabase Soft, (f) Trusoft, and (g) control (medium without eluate).

**Figure 4:**
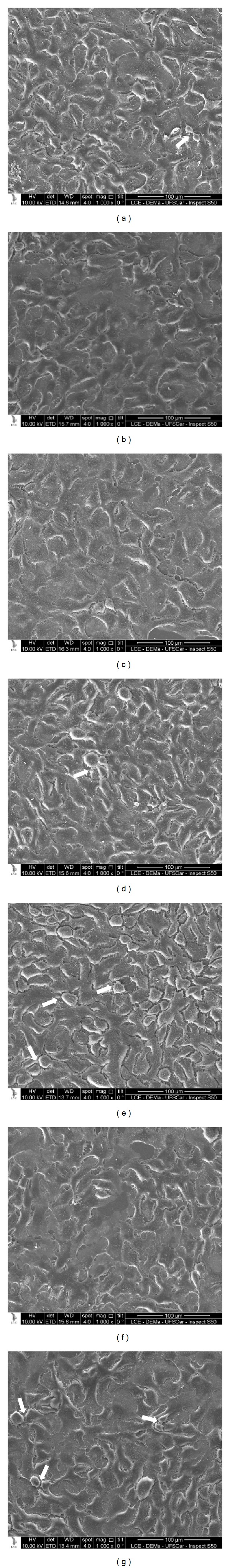
Panel of SEM micrographs (×1000) representative of the HaCat cells exposed to the 48 hour eluates from materials (a) Ufi Gel P, (b) Sofreliner S, (c) Coe Comfort, (d) Softone, (e) Durabase Soft, (f) Trusoft, and (g) control (medium without eluate).

The SEM photomicrographs in Figures 3(g) and 4(g) show that, for controls, large numbers of HaCat cells covered the surfaces of the glass substrate, some undergoing mitosis. It can also be observed that the typical polygonal cobblestone pattern of keratinocytes remained unchanged. Mitoses were also observed after exposure to the 24 hour eluates from Coe Comfort (Figure 3(c)), Durabase Soft (Figure 3(e)), and Trusoft (Figure 3(f)). Similar results were observed when HaCat cells were incubated with the 48 hour eluates. Normal cell morphology was maintained and mitoses were seen after exposure to Ufi Gel P (Figure 4(a)), Softone (Figure 4(d)), and Durabase Soft (Figure 4(e)) eluates.

**Figure 5:**
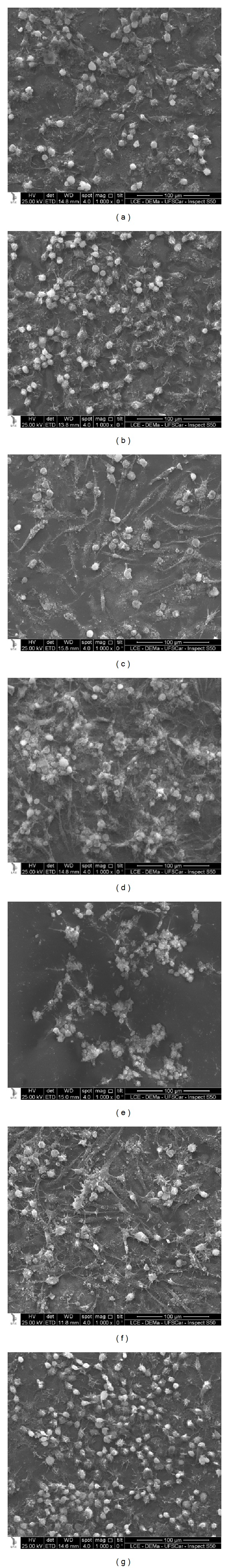
Panel of SEM micrographs (×1000) representative of the Raw 264.7 cells exposed to the 24 hour eluates from materials (a) Ufi Gel P, (b) Sofreliner S, (c) Coe Comfort, (d) Softone, (e) Durabase Soft, (f) Trusoft, and (g) control (medium without eluate).

**Figure 6:**
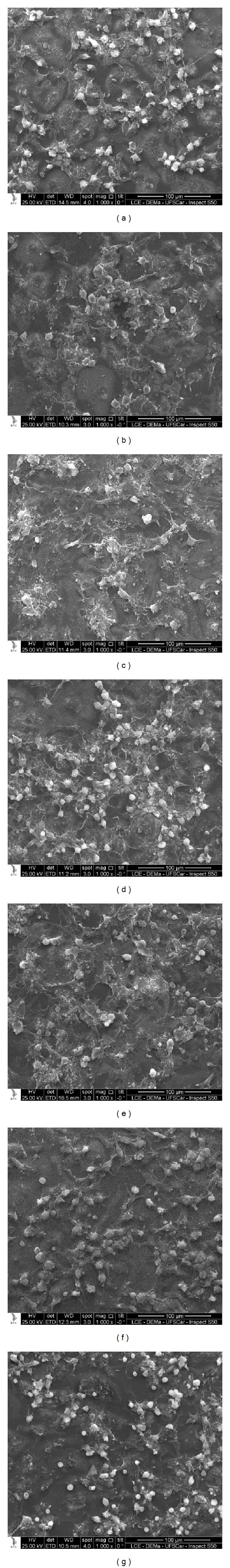
Panel of SEM micrographs (×1000) representative of the Raw 264.7 cells exposed to the 48 hour eluates from materials (a) Ufi Gel P, (b) Sofreliner S, (c) Coe Comfort, (d) Softone, (e) Durabase Soft, (f) Trusoft, and (g) control (medium without eluate).

Untreated RAW 264.7 cells (Figures 5(g) and 6(g)—controls) remained small and round, with a rather smooth surface and emitting short and scanty filamentous projections. After exposure to the 24 hour eluates from Coe Comfort and Durabase Soft (Figures 5(c) and 5(e), resp.), cell morphology was not well-defined, and remains of the cytoplasmic membranes of lethally damaged cells can be observed on the glass substrate. Figures 6(b), 6(c), and 6(e) show micrographs of RAW 264.7 macrophages after incubation with the 48 hour eluates from Sofreliner S, Coe Comfort, and Durabase Soft, with poorly defined cells and remnants of cytoplasmic debris.

The type of cell death (apoptosis or necrosis) as assessed by flow cytometry (Table 3) showed that, for L929 cells, the percentages of apoptotic cell death induced by the 24 hour eluates from Ufi Gel P and Sofreliner S and the 48 hour eluate from Durabase Soft were significantly higher than in controls (*P* = 0.021, *P* = 0.000, and *P* = 0.003, resp.). The percentages of L929 necrotic cells after exposure to the 24 and 48 hour eluates from all materials were higher than that of control (*P* < 0.02), except for Trusoft (*P* = 0.551). For HaCat cells, there were no statistically significant differences between the percentages of apoptosis or necrosis induced by the 24 or 48 hour eluates from all materials and their respective controls (*P* > 0.05).

### 3.2. Effect of Direct Contact on Cell Viability (MTT Assay)

Table 4 shows that, compared with the 24 hour period, all materials resulted in a significant decrease in cell viability after 48 h of direct contact with the L929 cells (*P* = 0.000). For both periods, Ufi Gel P and Sofreliner S promoted higher percentages of cell viability (*P* < 0.001 and *P* < 0.003, resp.), while the lowest percentages were obtained for Durabase Soft and Coe Comfort (*P* = 0.000 and *P* < 0.007, resp.). Softone showed an intermediate value, which was not significantly different from that of Trusoft at the 48 hour period (*P* = 0.055).

Similar results were observed for HaCat cells, and the percentages of cell viability at 24 h were significantly higher than those obtained at the 48 hour period (*P* = 0.006). The highest mean values were observed after direct contact of HaCat cells with Ufi Gel P, Sofreliner S, and Trusoft (*P* < 0.002, *P* < 0.006, and *P* < 0.001, resp.), and the lowest mean value was obtained for Durabase Soft (*P* < 0.002), regardless of the contact period (Table 4). Softone and Coe Comfort provided intermediate values in both periods.

For RAW 264.7 cells, a significant effect was observed only for the factor material, and no significant differences were found between the two periods (*P* > 0.05). Direct contact of RAW 264.7 cells with Ufi Gel P and Sofreliner S resulted in the highest percentages of cell viability (*P* = 0.000). The lowest mean percentages were found for Durabase Soft, Softone, and Coe Comfort (*P* = 0.000), while direct contact with Trusoft resulted in an intermediate value that was significantly different from those promoted for the other materials (*P* = 0.000).

## 4. Discussion

The immortalized cell lines L929, HaCat, and Raw 264.7 used in this study are sensitive to dental monomers and plasticizers that can be released from polymer materials. In addition, L929 fibroblasts are recommended by ISO 10993-5 for cytotoxicity tests [21], and HaCat cells are suitable substitutes for oral keratinocytes because they can be easily grown and passaged indefinitely [22]. Raw 264.7 cells present most of the functions of primary cultured macrophages [23], which are the first line of defense against potentially harmful substances. Due to their role in pathogenesis of inflammatory response and in the removal of cellular debris [24], macrophages are relevant for testing the biocompatibility of the soft liners that are commonly used in areas of ulcerated tissue or in the healing phase [1, 20].

The results from MTT tests showed that, in general, the materials were not cytotoxic to L929, HaCat, and RAW 264.7 cells, except the 48 hour eluates from Ufi Gel P, Coe Comfort, and Softone, which were slightly cytotoxic to the HaCat cells (cell viability values higher than 70%). Additionally, there were no significant differences among the materials for any of the three cell lines. Studies that evaluated the effects of 24, 48, and 96 hour eluates from silicone soft liners [5, 12, 13], among them Ufi Gel P, also showed no significant decrease in the L929 cell viability. Conversely, Krunić et al. [14] observed lower cytotoxicity for the silicone-based soft liners GC Reline Soft and Ufi Gel P, compared with the acrylic-based materials Flexacryl Lang, Lang Immediate, Vertex Soft, and Trusoft. For Ufi Gel P, the mean percentages of cell viability (MTT assay) ranged from 50 to 80%, which were lower than those obtained in the present study. These results may be related to the longer extraction period (30 day eluate) as well as the different cell line (HeLa) used by the authors [14].

Scanning electron microscopy (SEM) has been used to assess changes in cell morphology induced by toxic components released from dental materials [25]. The absence of significant reductions in cell viability after exposure to eluates from the materials does not exclude the possibility of damage to delicate cell structures. Thus, in the present study, microscopic analysis of cellular morphology was performed. In general, the number and morphology of L929 and HaCat cells attached to the glass substrate were similar to those observed for the control groups. However, for RAW 264.7 cells, the SEM micrographs revealed that cell morphology was not well-defined, with remnants of damaged cells, particularly after exposure to the eluates from Durabase Soft and Coe Comfort. Exposure of L929 cells to the 48 hour eluate from Coe Comfort also altered their morphology, with poorly defined cellular limits.

It is known that the pattern of cell death may play a role in determining the cytotoxicity and irritation potential of dental materials, since apoptotic cells are removed by phagocytosis and trigger discrete tissue damage compared with the severe inflammation observed in necrosis [26]. In the present study, flow cytometry demonstrated an increase in percentages of apoptosis and necrosis only for L929 cells. These findings suggested that the tested eluates induced higher toxic effects on L929 cells (fibroblasts) than on HaCat cells (keratinocytes). Nevertheless, the results could imply that the numbers of remaining cells are still sufficient to have mitochondrial activity similar to that of the controls (cells without contact with the eluates), as confirmed by the cell viability (MTT assay).

MTT assays performed with direct contact between the soft liners and the cells determined significant differences among the materials that were not detected in the tests performed with eluates. It is possible that, in the direct contact tests, the presence of the specimen during testing prevented the evaporation/degradation of the components released [16], which may have occurred during the extraction periods for the preparation of eluates. For both direct contact time periods (24 and 48 h), the silicone-based materials Sofreliner S and Ufi Gel P were less cytotoxic to the L929, HaCat, and RAW 264.7 cells, while the acrylic-based reliner Durabase Soft exerted greater effects. The tissue conditioners Coe Comfort and Softone were also cytotoxic, particularly to RAW 264.7 cells. Trusoft exhibited an intermediate cytotoxicity to L929 and HaCat cells and was not significantly different from silicone-based liners. However, for RAW 264.7 cells, Trusoft resulted in lower percentages of cell viability. Silicone-based liners are similar to polyvinylsiloxane-based impression materials [1] and polymerize by an addition reaction with no by-products, such as alcohol. It has been found that, although components such as monomers and phthalic plasticizers were released by soft liners, the silicone-based materials, including Ufi Gel P, were generally more stable, releasing smaller quantities than the acrylic-based liners [7, 8]. Thus, it can be supposed that Ufi Gel P and Sofreliner S exerted less-pronounced cytotoxic effects on the three cell lines due to a lower release of residual components.

The cytotoxic activity of Durabase Soft in the three cell lines evaluated may be related with its composition. The powder contains benzoyl peroxide (BP), used as an initiator of the polymerization reaction [27, 28]. BP has a toxic effect which is mediated by its free radical derivatives, resulting from the cleavage of the peroxide bond [27, 28] during polymerization reaction. Free radicals put the cells under toxic conditions [29] and may influence lipid metabolism, affect viability [28], and cause an increase in multinucleation and extensive vacuolization of cells [27]. Babich et al. [27] found that BP was approximately 10 times more toxic than hydrogen peroxide and about 250 times more toxic than its final degradation product, the benzoic acid. With regard to the liquid component, Durabase Soft contains methylmethacrylate (MMA), which can be released from acrylic-based soft liners in greater amounts than from silicone-based materials [7, 8]. Although the processes underlying the toxicity of monomers have not been fully elucidated, some mechanisms have been reported, such as DNA damage [29], injury to the lipid bilayer [30], and changes in the mobilization of calcium ions (Ca^2+^), which play an important role in many pathways of cellular signal transduction [30]. Another proposed mechanism is the reduction or depletion of glutathione, a direct scavenger of toxic radicals [29, 31], caused by reactive oxygen species (ROS) generated by resin materials or through the reaction of *α*,*β*-unsaturated methacrylate esters, including MMA, with glutathione via the Michael addition [32]. After glutathione depletion, the increase in ROS results in oxidative stress that can lead to DNA damage, cell cycle delay, and, eventually, cell death [29, 31, 33]. In general, acrylates and dimethacrylates show a higher affinity for glutathione and higher toxicity than methacrylates such as MMA, which has only one methacrylate group [32, 34]. Nevertheless, it can be supposed that the presence of MMA in Durabase Soft liquid contributed, at least in part, to the cytotoxic effects observed in the present study. Moreover, MMA can decompose via hydrolysis in methacrylic acid (MA) [35], which also has a cytotoxic effect [36], altering both cellular metabolism and DNA synthesis [37].

The materials evaluated also contain plasticizers such as di-butyl phthalate (DBP) in Durabase Soft and Softone, benzyl butyl phthalate (BBP) in Trusoft, and the phthalate ester-free plasticizer benzyl benzoate (BB) in Coe Comfort. The toxicity of phthalates is caused by their metabolite methoxyacetic acid (MAA), through mechanisms that involve ROS generation and DNA and mitochondrial damage [38]. The fat-soluble compound BB can enter cells through the membrane and act on the mitochondria, inhibiting respiration and producing ROS, which can attack polyunsaturated fatty acids and induce lipid peroxidation [39]. The exposure of L929 cells to DBP for 24 h [37] and macrophages RAW 264.7 to plasticizers BBP and DBP for 60 min [40] led to a reduction in DNA synthesis, cell metabolism, and viability. Thus, it is likely that the cytotoxic effects observed here for Durabase Soft, Softone, Coe Comfort, and, to a lesser extent, Trusoft are related, at least in part, to the presence of plasticizers in their compositions. Other studies also found that the direct contact between different cells and tissue conditioners, including Coe Comfort, resulted in cytotoxic effects, such as zones of growth inhibition, cell lysis [15], and reduced cell viability [41]. Conversely, Kostić et al. [18], who evaluated soft liners in direct contact with HeLa cells for 24 h, found that Trusoft yielded an average cell viability of 78.7%, which is higher than those observed here for HaCat and RAW 264.7 cells and lower than that recorded for the L929 cells. The different cell lines used may have contributed to these dissimilar results. In the present study, in general, the effects of direct contact of the tested materials were more pronounced on HaCat and RAW 264.7 cells.

The greater effects observed in this study for the direct contact between cells and the tissue conditioner Coe Comfort could also be related to the lower molecular weight of the plasticizer BB (212.25) [6] compared with that of DBP (278.34) [6, 40] and BBP (312.36) [40], which are contained in Softone and Trusoft, respectively. Plasticizers with higher molecular weight have a lower release from soft liner materials [6]. Hong et al. [42] found that the amount of plasticizer BB released from Coe Comfort was significantly higher than those of plasticizers BBP and DBP released from the other materials, in both periods (1 and 14 days).

Although the results obtained from* in vitro* studies cannot be directly extrapolated to clinical situations, it is important to consider whether the changes observed in the present investigation are cumulative and become increasingly pronounced with time. This could make the cells more susceptible to subsequent challenges, such as direct contact with newly applied soft liner materials. It is important to note that, due to a progressive loss of plasticizers and alcohol, the soft liners need to be replaced at regular intervals. Although these replacements will prevent trauma and colonization of the material by microorganisms, they are performed directly in the mouth, increasing the exposure of tissues to the leached components, which, even in low concentrations, can lead to chronic adverse effects on the oral mucosa. Even if not causing acute cytotoxicity, the continuous release of such substances may compromise tissue homeostasis and repair, which are particularly important given that some soft liner materials are applied over inflamed supporting tissues and during the healing phase in immediate dentures or dental implants. Based on the results of this study, silicone-based materials seem to be the most suitable in terms of biocompatibility.

## 5. Conclusions

It can be concluded that the 24 and 48 hour eluates from all materials were not cytotoxic to the three cell lines tested. However, alterations on the morphology of the macrophages RAW 264.7 cells were induced. It was also observed that the fibroblasts and keratinocytes showed different sensitivities when exposed to the 24 and 48 hour eluates from the soft liner materials tested, with fibroblasts showing higher percentages of cell necrosis for both extraction periods. Moreover, in the direct contact tests, effects on cell viability were more pronounced, particularly for the acrylic-based reliners (Durabase Soft and Trusoft) and the tissue conditioners (Coe Comfort and Softone). All these findings are of importance because the soft liners are often applied on in areas of ulcerated tissue or after surgery, where both macrophages and fibroblasts play important roles in the healing process. The silicone-based materials, Ufi Gel P and Sofreliner S, caused less reduction in cell viability when in direct contact with the three cell lines tested, for both periods (24 and 48 hours). Taken together, these results indicated that the silicone-based soft liners may have a more suitable biological behavior and might reduce the risk of adverse effects in clinical use.

## Figures and Tables

**Table 1:** Materials evaluated in this study.

Product	Type	Manufacturer	Powder/liquid ratio	Composition (manufacturer supplied)	Batch number	Polymerization/gelation time
Durabase Soft	Plasticized acrylic resin soft liner	Reliance Dental Mfg Co., Alsip, IL, USA	1 g/0.83 mL	Powder-PEMA and benzoyl peroxide Liquid-MMA and DBP	29549	15 min

Trusoft	Plasticized acrylic resin soft liner	Bosworth Co, Skokie, IL, USA	1.06 g/1.15 mL	Powder-PEMA Liquid-Benzyl butyl phthalate (plasticizer) and ethyl alcohol	0904-137	6 min

Ufi Gel P	Silicone-based soft liner	Voco, Cuxhaven, Germany	Base and catalyst in a 1 : 1 ratio	Base-modified polydimethylsiloxane (A-silicone) Catalyst-platinum catalyst	1009051	10 min

Sofreliner S	Silicone-based soft liner	Tokuyama Dental Corp., Tokyo, Japan	Autodispensing system	Polyorganosiloxane Silicone resin powder Silica, amorphous	035E50	5 min

Softone	Tissue conditioner	Bosworth Co, Skokie, IL, USA	1.03 g/1.1 mL	Powder-PEMA Liquid-DBP and ethanol	0906-231	8 min

Coe Comfort	Tissue conditioner	GC America Inc., Alsip, IL, USA	1 g/0.83 mL	Powder-PEMA and zinc undecylenate Liquid-Benzyl Benzoate, cotton seed oil, ethanol, acetyl tributyl citrate, methyl salicylate, and peppermint oil	1006032	10 min

PEMA: poly (ethyl methacrylate); MMA: methyl methacrylate; and DBP: di-butyl phthalate.

**Table 2:** Means and standard deviations (SD) of cell viability (% of controls) after cells exposure to 24 or 48 hour eluates from materials.

Material	Cell line					
	L929		HaCat		RAW 264.7	
	Eluate period		Eluate period		Eluate period	
	24 h	48 h	24 h	48 h	24 h	48 h
Durabase Soft	78.9 (27.6)	94.5 (18.8)	98.1 (10.6)	88.4 (8.1)∗	90.1 (27.9)∗	85.8 (10.7)
Trusoft	84.8 (33.2)	110.5 (17.1)	109.5 (4.2)	81.0 (7.5)∗	104.4 (7.7)	94.7 (18.2)
Ufi Gel P	107.5 (13.1)	118.8 (14.0)	89.2 (19.6)	72.0 (14.4)∗	99.9 (8.6)	103.5 (8.5)
Sofreliner S	105.6 (5.9)	110.4 (9.7)	91.8 (7.1)	83.0 (15.5)∗	92.2 (9.3)	95.6 (28.2)
Softone	88.3 (7.5)	89.4 (13.0)	84.6 (21.8)	71.8 (25.3)∗	85.8 (20.4)	82.6 (15.7)
Coe Comfort	100.8 (9.7)	108.1 (13.1)	81.6 (22.9)	75.2 (32.9)∗	85.0 (10.8)	88.2 (16.3)

No statistically significant differences were found among the materials, regardless of cell line or eluate period (*P* > 0.05).

∗There was a statistically significant difference between the two periods (24 and 48 hour eluates).

**Table 3:** Geometric means and standard deviations (SD) of percentages of apoptosis and necrosis after exposure of L929 and HaCat cells to 24 or 48 hour eluates from materials.

Eluate period	Material	Cell line			
		L929		HaCat	
		Apoptosis (%)	Necrosis (%)	Apoptosis (%)	Necrosis (%)
24 h	Durabase Soft	3.41 (2.31)^ab^	20.42 (1.39)^b^	7.94 (1.58)^a^	2.54 (2.24)^cd^
	Trusoft	2.89 (2.02)^ab^	12.52 (1.72)^ab^	8.59 (1.83)^a^	2.42 (2.16)^cd^
	Ufi Gel P	12.73 (2.24)^bc^	18.44 (1.45)^b^	5.94 (1.58)^a^	3.32 (2.46)^cd^
	Sofreliner S	25.08 (2.17)^c^	22.51 (1.41)^b^	5.68 (1.31)^a^	2.03 (1.18)^cd^
	Softone	2.31 (1.98)^a^	24.78 (1.47)^b^	4.76 (1.29)^a^	1.72 (2.04)^c^
	Coe Comfort	1.30 (3.70)^a^	14.85 (1.79)^b^	7.11 (1.36)^a^	4.32 (1.33)^d^
	Control	2.39 (2.35)^a^	6.88 (1.91)^a^	7.17 (1.33)^a^	2.03 (2.35)^cd^

48 h	Durabase Soft	12.15 (3.08)^bc^	17.91 (1.71)^b^	6.96 (2.07)^b^	1.17 (1.85)^ab^
	Trusoft	1.96 (2.54)^a^	10.74 (1.82)^ab^	12.02 (2.48)^b^	1.48 (1.65)^ab^
	Ufi Gel P	2.91 (2.52)^ab^	13.44 (1.87)^b^	8.70 (2.33)^b^	2.31 (1.80)^ab^
	Sofreliner S	2.43 (3.25)^a^	13.83 (1.70)^b^	6.53 (1.98)^b^	1.99 (1.58)^ab^
	Softone	2.41 (1.64)^a^	15.09 (1.70)^b^	8.09 (2.25)^b^	1.24 (1.79)^a^
	Coe Comfort	4.31 (1.95)^ab^	16.04 (2.15)^b^	12.73 (1.51)^b^	2.34 (2.40)^b^
	Control	1.72 (2.47)^a^	10.05 (1.32)^a^	8.20 (1.82)^b^	1.59 (1.90)^ab^

For each cell line, the same superscripted lowercased letters indicate no statistically significant differences in columns (*P* > 0.05).

**Table 4:** Means and standard deviations (SD) of cell viability (% of controls) after direct contact of L929, HaCat, and RAW 264.7 cells with the materials for 24 or 48 h.

Material	Cell line					
	L929		HaCat		RAW 264.7	
	Eluate period		Eluate period		Eluate period	
	24 h	48 h	24 h	48 h	24 h	48 h
Durabase Soft	15.8 (3.9)^Aa^	13.9 (8.0)^Ba^	8.2 (6.0)^Aa^	1.7 (1.5)^Ba^	5.8 (4.4)^Aa^	5.4 (8.7)^Aa^
Trusoft	97.9 (17.7)^Ac^	51.2 (8.4)^Bbc^	65.2 (20.1)^Acd^	61.3 (25.4)^Bcd^	57.4 (17.0)^Ab^	37.7 (19.0)^Ab^
Ufi Gel P	99.3 (14.6)^Ac^	71.6 (12.4)^Bc^	89.0 (24.1)^Ad^	69.7 (4.9)^Bd^	84.0 (6.9)^Ac^	106.2 (10.0)^Ac^
Sofreliner S	91.4 (7.9)^Ac^	73.9 (3.7)^Bc^	86.6 (15.3)^Ad^	63.6 (8.3)^Bd^	89.2 (11.5)^Ac^	97.2 (17.0)^Ac^
Softone	60.6 (15.4)^Ab^	46.1 (21.8)^Bb^	44.1 (26.7)^Abc^	39.3 (20.9)^Bbc^	16.3 (8.9)^Aa^	15.3 (18.6)^Aa^
Coe Comfort	31.2 (19.8)^Aa^	21.8 (21.0)^Ba^	37.0 (21.7)^Aab^	6.4 (4.2)^Bab^	7.9 (8.1)^Aa^	4.8 (6.2)^Aa^

For each cell line, the same superscripted capital letters indicate no statistically significant differences in rows, and the same superscripted lowercased letters indicate no statistically significant differences in columns (*P* > 0.05).
